# Epidemiological Characteristics and Mortality Risk Factors Comparison in Dialysis and Non-Dialysis CKD Patients with COVID-19—A Single Center Experience

**DOI:** 10.3390/jpm12060966

**Published:** 2022-06-13

**Authors:** Andrei Niculae, Ileana Peride, Ana-Maria Nechita, Lucian Cristian Petcu, Mirela Tiglis, Ionel Alexandru Checherita

**Affiliations:** 1 Clinical Department No. 3, “Carol Davila” University of Medicine and Pharmacy, 050474 Bucharest, Romania; niculaeandrei@yahoo.com (A.N.); al.checherita@gmail.com (I.A.C.); 2 Department of Nephrology and Dialysis, “St. John” Emergency Clinical Hospital, 042122 Bucharest, Romania; distimia_28@yahoo.com; 3 Department of Biophysics and Biostatistics, Faculty of Dentistry, “Ovidius” University, 900684 Constanta, Romania; crilucpetcu@gmail.com; 4 Clinical Department No. 14, “Carol Davila” University of Medicine and Pharmacy, 050474 Bucharest, Romania

**Keywords:** COVID-19, CKD, chronic dialysis, biomarkers, mortality

## Abstract

(1) Background: Despite some controversies between studies, chronic kidney disease (CKD) has a negative impact on COVID-19 outcomes, with patients presenting a higher mortality risk than in the general population. Studies have shown an association between COVID-19 severe cases and different inflammatory biomarkers. The aim of this study was to emphasize the epidemiological characteristics of CKD patients diagnosed with COVID-19 and to determine if the risk of mortality, and the severity of this infection might be influenced by different parameters. (2) Methods: Our retrospective study included CKD patients with COVID-19—362 in the non-dialysis group and 132 in the dialysis group. (3) Results: There were significant statistical differences between our groups regarding age (*p* < 0.001), hemoglobin (*p* < 0.001), interleukin-6 (*p* < 0.001), serum albumin (*p* = 0.016), procalcitonin (*p* = 0.002), ferritin (*p* < 0.001), and of course serum creatinine (*p* < 0.001). Even if the risk of death was higher in the dialysis group (Exp(b) = 1.839), the survival proportions were similar in both groups. (4) Conclusions: High values of hemoglobin, serum creatinine, and LDH at admission, age, length of hospital stay ≤ 10 days, and a pulmonary impairment > 25% are responsible for an adverse outcome in non-dialysis and dialysis patients diagnosed with COVID-19.

## 1. Introduction

COVID-19 was declared a global pandemic by the World Health Organization (WHO) on 11th of March 2020 [1,2] and according to the reported data from 2021, more than 233 million people were diagnosed with COVID-19 and 4.7 million died [3,4].

Several studies have shown that different pathological conditions, such as chronic kidney disease (CKD) [5], diabetes mellitus [6], hypertension, neoplasia, and chronic respiratory diseases, have a negative impact on the evolution of COVID-19—more severe cases and increased mortality [7].

CKD, with an estimated worldwide prevalence of 9–12% [8], triggers high morbidity and mortality, especially in the advanced stages [9]. Therefore, since 2020, different trials and systemic reviews have been performed in order to determine the influence of preexistent renal impairment on the progression and outcome of COVID-19 [7], and despite some controversies between studies, CKD has a negative impact on COVID-19 outcome [10]. It has been reported that there is an associated mortality risk that is 3 to 4-fold higher in chronic dialysis and renal transplanted patients infected with SARS-CoV-2 than in the general population [11,12]. Furthermore, it is considered that renal transplanted patients present a higher risk of mortality, probably due to their long-term immunosuppressive therapy that can contribute to an increased frail profile and the lack of unified treatment protocol when COVID-19 is associated. Different therapies were proposed including the use of tocilizumab at standard dosage before the development of pulmonary impairment and inflammation activation seen during the infection with SARS-CoV-2, but specific controlled trials are required to validate these findings [13].

The pathophysiological mechanisms involved at the onset of COVID-19 and their influence on kidney function are seen as a major concern, especially because it was noticed in critically ill patients that the presence of hypoxia, hypotension, low cardiac output, and the requirement of invasive mechanical ventilation and the use of nephrotoxic agents (i.e., vancomycin, colistin, and aminoglycosides) were factors with a direct impact on renal function [14].

At the onset of COVID-19, a series of molecular pathways are incriminated, such as the renin–angiotensin system, immune response and related pathways, signal transduction, and cellular process. It has been documented that angiotensin-converting enzyme 2 (ACE2) plays the role of the receptor for the SARS-CoV-2 virus: the transmembrane spike glycoprotein (S) of the SARS-CoV-2 virus binds with ACE2 receptors (the N-terminal segment) at its receptor-binding domain (RBD). This interaction is facilitated by TMPRSS2 (transmembrane serine protease 2), which acts as a primer for the SARS-CoV-2 S protein. Because there were cases of infection with SARS-CoV-2 presenting mutations of the RBD, resulting in a superior resistance to neutralize the antibodies and increase the affinity to ACE2, different monoclonal antibodies were proposed as viable treatment options (i.e., bamlanivimab, bamlanivimab/etesevimab, regdansimab, casirivimab/imdevimab, sotrovimab, etc.) [15]. It should be emphasized that RAS consists of two pathways [15]:

- The ACE/Ang II/AT1R pathway (angiotensin-converting enzyme/angiotensin II/angiotensin II type 1 receptor)—incriminated in the development of lung injury, cell proliferation, inflammation, etc.
- The ACE2/Ang 1-7/MasR pathway (angiotensin-converting enzyme 2/angiotensin 1-7/Massey receptor)—with a protective role on the respiratory system.

**Figure 1:**
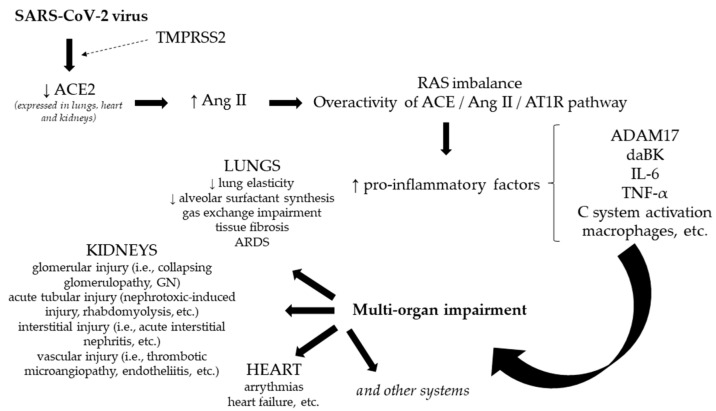
The link between SARS-CoV-2 infection and multi-organ impairment. Notes: ACE2—angiotensin-converting enzyme 2; ADAM17—a disintegrin and metalloproteinase 17 protease; Ang II—angiotensin II; ARDS—acute respiratory distress syndrome; TMPRSS2—transmembrane serine protease 2; AT1R—angiotensin II type 1 receptor; C system—complement system; daBK—des arginine9-bradikinine; GN—glomerulonephritis; IL-6—interleukin-6; RAS—renin–angiotensin system; TNF-α—tumor necrosis factor alpha.

Once the S protein of the SARS-CoV-2 virus interacts with ACE2, membranal ACE2 decreases and favors the increase in Ang II, and consequently an elevated activity of the ACE/Ang II/AT1R pathway, producing a significant imbalance between the two pathways of RAS. Ang II overactivity is responsible for the activation of macrophages, interleukin-6, tumor necrosis factor alpha, and other cytokines, and once AT1R is activated, the stimulation of ADAM17 (a disintegrin and metalloproteinase 17) protease is noticed, which is incriminated in further intracellular degradation and inflammation (therefore, therapies targeting ADAM17 are being considered as efficient). In addition, the complement system is activated, contributing to a further inflammatory state. Furthermore, it was observed that ACE2 depletion favored the stimulation of a pulmonary inflammatory factor—daBK (des arginine9-bradikinine), responsible for BK receptor B1 activation and, consequently, the onset of a cytokine storm and even acute respiratory distress syndrome (ARDS). ACE2 down-regulation leads to a decreased lung elasticity, alveolar surfactant synthesis, gas exchange impairment, and finally to fibrosis, as well. Considering the deleterious impact of ACE/Ang II/AT1R pathway activation, along with the beneficial effects of the ACE2/Ang 1-7/MasR pathway in lung protection, several therapeutic strategies were recommended, such as recombinant human ACE2 (rhACE2), Ang 1-7 analogs, a combination of rhACE2 and remdesivir, etc., which could enhance the activity of ACE2, and consequently to combat the progression of COVID-19 [15]. Furthermore, considering that the expression of ACE2 could be noted, not only in the lungs, but also in the renal and cardiac systems, it could represent a plausible explanation for the correlation between the infection with SARS-CoV-2 and these two systems, including the presence of various manifestations, such as arrhythmias [16], acute cardiac, and renal injury [14,15], potentially inducing the onset of a secondary cardiorenal syndrome. Therefore, the treatment of COVID-19, especially in critically ill patients, should include a pluri-management approach. In Figure 1 are represented the underline mechanisms involved in COVID-19, linking SARS-CoV-2 infection to multi-organ impairment.

Additionally, there are studies that have shown the association between COVID-19 severe cases and different biomarkers, such as lymphopenia, thrombocytopenia, D-dimer, serum creatinine, lactate dehydrogenase, C-reactive protein, procalcitonin, creatinine kinase, cardiac troponin, etc. [17,18,19,20].

Therefore, the aim of our study was to determine the prognosis of CKD patients (dialysis and non-dialysis) diagnosed with COVID-19 since day-1 from admission, based on their epidemiological characteristics and several specific biomarkers, and, in future studies, the possibility to develop an adequate score of severity correlated with the risk of mortality and length of hospital stay in this population group.

## 2. Materials and Methods

Our retrospective study (approved by the Hospital Ethics Committee, no. 1571/26 January 2022) included CKD patients (dialyzed or not) diagnosed with moderate and severe forms of COVID-19 admitted to an emergency clinical hospital, between 1 November 2020 and 31 December 2021. The diagnosis of COVID-19 was established based on positive results of rapid antigen tests or RT-PCR (reverse transcription-polymerase chain reaction), in accordance with our national protocol issued by the National Center of Surveillance and Control for Contagious Diseases, which regulated that all symptomatic persons (i.e., cough, fever, acute respiratory insufficiency, anosmia, ageusia, etc.) or direct contacts with confirmed cases should be tested. Additionally, chronic dialyzed patients should be tested twice a month.

The design of the study included the following features, collected at the time of admission: age, gender, patient’s environment, the medical department where they were admitted, the presence or absence of diabetes mellitus, obesity, dialysis, the degree of pulmonary impairment, and also different bioumoral parameters specific to CKD and inflammation (Table 1). Related to dialysis, we included all patients with a chronic dialysis diagnostic existing in the informatic database of the hospital, recorded by each physician in the patients’ discharge medical report.

The degree of pulmonary impairment was determined by a specialized medical team, based on computer tomography images (the presence of multiple ground-glass opacities), and the results were grouped into 5 grades of severity depending on the affected surface area:

- Without impairment = grade 1;
- ≤25% = grade 2;
- 26–50% = grade 3;
- 51–75% = grade 4;
- 76–100% = grade 5.

Additionally, data regarding the length of hospital stay (LOS), discharge status, and types of oxygen therapies (high-flow oxygen therapy, invasive and non-invasive mechanical ventilation) were collected.

The patients were selected using the informatic database of the hospital, applying the following filters:

- Time period: between 1 November 2020 and 31 December 2021;
- Diagnosis of COVID-19 and CKD. The diagnosis of CKD was based on the main and secondary diagnosis (according to our national protocol of diagnosis index) related to this pathology, as it was recorded by each physician in the patients’ discharge medical report.

After all the filters were applied, the patients included in our study were divided into 2 groups: non-dialysis group and dialysis group, and the assessed variables were compared between these groups.

### Statistical Analysis

The statistical analysis was performed using IBM SPSS statistics software version 23 and MedCalc 14.8.1. Data are presented as mean ± standard deviation (SD) for continuous variables in case of symmetric distributions, median, and IQR (Interquartile range P75-P25) for continuous variables in case of skewed distributions, or as percentages for categorical variables. The normality of the continuous data was estimated with Kolmogorov–Smirnov Tests of Normality. For hypothesis testing, the following tests were used: Independent Samples Test, Independent Samples Mann–Whitney U Test, and Median Test. Logistic regression was used to find the best fitting model to describe the relationship between the dependent variable (Deceased: Yes/No) and a set of independent variables. The factor Exp(b) is the “adjusted” odds ratio (OR) for the independent variable and it gives the relative amount by which the odds of the outcome increase (OR greater than 1) or decrease (OR less than 1) when the value of the independent variable is increased by 1 unit. We followed recommendations based on the work of Peduzzi et al. (1996) [21] to calculate the minimum sample size required for prediction model development. The significance level α was set at 0.05.

## 3. Results

**Figure 2:**
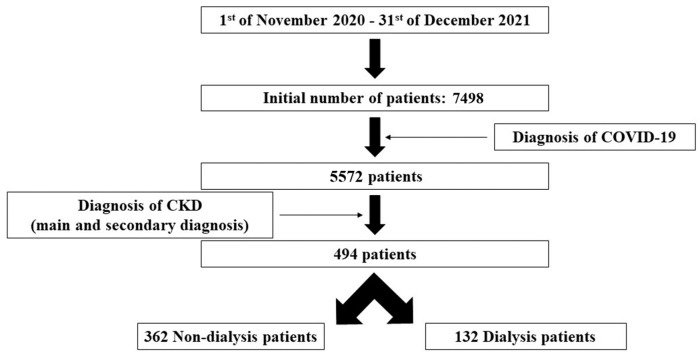
Design of the study. Notes: CKD—chronic kidney disease.

After applying the filters indicated in the methodology, 494 CKD patients diagnosed with COVID-19 were included in the study (during the mentioned time period, a total of 7498 patients were admitted to our hospital, 5572 being diagnosed with COVID-19)—Figure 2. All the patients received our standard national protocol regimen for the infection with SARS-CoV-2 as stipulated by the Romanian Health Ministry, but no data were available regarding the customized therapy for each patient in the informatic database of the hospital. Additionally, only 29 of them were vaccinated, an amount that could not be considered statistically significant for further assessment. Furthermore, the dialysis group included also only one patient undergoing peritoneal dialysis (without statistical significance).

The characteristics of our selected patients in both groups are described in Table 2.

Our findings indicated that there were significant statistical differences between the two groups regarding age (*p* < 0.001), hemoglobin level (*p* < 0.001), interleukin-6 concentration (*p* < 0.001), serum albumin level (*p* = 0.016), procalcitonin concentration (*p* = 0.002), ferritin level (*p* < 0.001), and of course serum creatinine concentration (*p* < 0.001), as we compared non-dialysis vs. dialysis patients. Between the non-dialysis and dialysis groups, no associations were found related to gender, the presence of diabetes mellitus, obesity, the grade of pulmonary impairment, or mortality. The modality of oxygen therapy appears to be similar in both groups, except the non-invasive mechanical ventilation that was more likely to be used in the dialysis group (*p* = 0.043).

As expected, most of our patients were admitted by internal medicine departments (i.e., Nephrology, Cardiology, or Internal medicine), and fewer by the surgical units.

Even if there were no differences between the two groups regarding mortality, a higher percentage of deceased patients was noticed in the dialysis group (43.9% vs. 42%). Therefore, in order to highlight the potential parameters that could influence the risk of mortality, we applied logistic regression, using the Backward method and Wald test to test the significance of the coefficients.

First, we evaluated the influence of the non-parametric variables on mortality, and initially, we introduced in our model as independent parameters: age, length of hospital stays, gender, grade of pulmonary impairment, diabetes mellitus, obesity, non-dialysis, and dialysis. Except diabetes and obesity that were excluded from the model (*p* = 0.176, *p* = 0.932, respectively), the rest of the variables seemed to present a significant influence on the risk of mortality.

Depending on the assessed group, our logistic regression was based on the following equations:

- Dialysis group

Logit (*p*) = −6.205 + 0.049 × age + 2.027 × length of hospital stay + 0.496 × gender + 2.038 × grade of pulmonary impairment + 0.609 × dialysis group

- Non-dialysis group

Logit (*p*) = −5.595 + 0.049 × age + 2.027 × length of hospital stay + 0.496 × gender + 2.038 × grade of pulmonary impairment−0.609 × non-dialysis group

For a better understanding, in Table 3 and Table 4 we described the adjusted odds ratio (Exp(b)) for each of the analyzed parameters (independent variables) that gives the relative amount by which the odds of the outcome (mortality occurrence) increase or decrease, in the dialysis group and non-dialysis group, respectively.

When analyzing the obtained results in both groups, we noticed that the odds (OR) for a positive outcome (mortality occurrence) were 7.590 times higher in cases where length of hospital stay was ≤10 days than in cases where length of hospital stay was >10 days. Furthermore, the risk was 1.642 times higher for males than female patients, 7.675 times higher for a grade of pulmonary impairment >25% than for a grade of pulmonary impairment <25%, and 1.839 times higher for the dialysis group than the non-dialysis group. In addition, when age increases by 1 unit (year), with all other factors remaining unchanged, then the odds will increase by a factor of 1.051.

Based on these results, for a male patient with the following characteristics: length of hospital stays (≤10 days), grade of pulmonary impairment (>25%), and belonging to the dialysis group or non-dialysis group, respectively, the probability of death, according to age (years) are presented in Table 5 and Table 6. For example, a 50 years old dialysis male patient with the above-mentioned characteristics presents a probability of death of 0.8046 (80.46%), and a non-dialysis patient has a probability of 0.6965 (69.65%).

To evaluate the predictive accuracy of our logistic regression models, ROC curve analysis was performed on the predicted probabilities calculated by the models and the dependent variable used in the logistic regression—area under the ROC curve AUC = 0.834 > 0.5 and *p* < 0.001 indicating a good logistic regression model.

Furthermore, we evaluated the influence of the parametric variables on the risk of mortality. Initially, we introduced—age, length of hospital stays, and the following biomarker concentrations (assessed at the admission): hemoglobin, serum creatinine, serum urea, LDH, glycemia, glycosylated hemoglobin, IL-6, CRP, quantitative D-dimer, procalcitonin, ferritin, ESR, and fibrinogen.

Serum urea (*p* = 0.898), glycemia (*p* = 0.509), glycosylated hemoglobin (*p* = 0.207), IL-6 (*p* = 0.902), CRP (*p* = 0.194), quantitative D-dimer (*p* = 514), procalcitonin, (*p* = 0.891), ferritin (*p* = 0.446), ESR (*p* = 0.173), and fibrinogen (*p* = 0.331) were excluded from the model.

In Table 7 we described the adjusted odds ratio (Exp(b)) for each of the analyzed parameters (independent variables) that gives the relative amount by which the odds of the outcome (mortality occurrence) increase or decrease.

When analyzing our findings, we observed that the odds (OR) for a positive outcome (mortality occurrence) were 10.643 times higher in cases where length of hospital stay was ≤10 days than in cases where length of hospital stay was >10 days. Furthermore, the risk was 1.260 times higher in cases of increased hemoglobin concentration, 1.165 times higher for elevated serum creatinine level, and 1.003 times higher for increased LDH activity. In addition, when age increases by 1 unit (year), with all other factors remaining unchanged, then the odds will increase by a factor of 1.048.

Similarly, to evaluate the predictive accuracy of our logistic regression model, ROC curve analysis was performed on the predicted probabilities calculated by the model and the dependent variable used in the logistic regression—area under the ROC curve AUC = 0.806 > 0.5 and *p* < 0.001 indicating a good logistic regression model.

Related to LOS and the degree of pulmonary impairment, we considered only ≤10 days of hospitalization and >25% pulmonary impairment to be significant, because when evaluating LOS and the grade of the pulmonary impairment we noticed that these values could influence the risk of mortality. When applying the odds ratio for the grade of pulmonary impairment (>25%/<25%) the following result was obtained: 6.972 with a 95% CI of 4.549 to 10.686. It means that for patients with a pulmonary impairment, >25% present a mortality risk of 6.972 higher than in patients with an impairment of less than 25% (as the 95% confidence interval does not contain the value one, and the result is higher than one).

**Figure 3:**
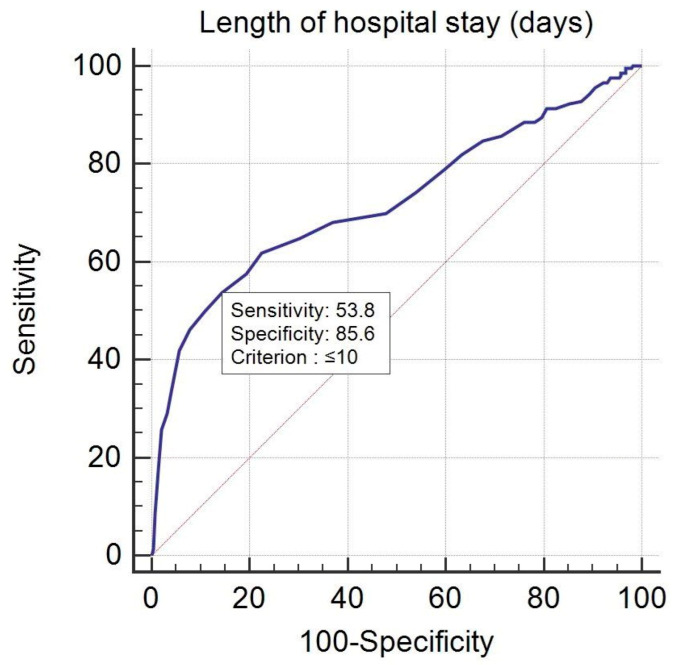
ROC curve analysis for the length of hospital stays.

Furthermore, when evaluating the length of hospital stay influence on the probability of mortality (deceased or not) using ROC curve analysis we obtained a *p* value < 0.001 that highlighted that the area under the ROC curve was significantly different from 0.5. Therefore, there was evidence that the variable length of hospital stay (days) had the ability to distinguish between the patients who died or not (A = 0.723, Youden index J = 0.3937, Se = 53.81%, Sp = 85.56%). The threshold or criterion value was ≤10 days: a sensitivity of 53.81 (95% CI of 46.8 to 60.7) and a specificity of 85.56 (95% CI of 80.9 to 89.4) (Figure 3).

Based on these results, comparing the patients with a grade of pulmonary impairment >25%, 45.71% died less than 10 days of hospitalization (LOS ≤ 10 days), and 36.67% after 10 days from the admission. Related to deceased patients with a grade of pulmonary impairment >25% and LOS ≤ 10 days, 41.43% of them required invasive mechanical ventilation (only 34.29% of the deceased patients after 10 days of hospitalization with a grade of pulmonary impairment >25% required this form of oxygen therapy).

**Figure 4:**
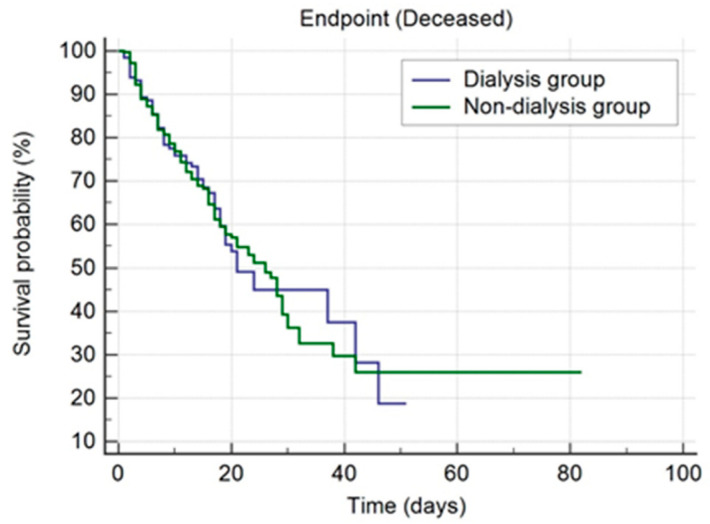
The curves of survival for non-dialysis and dialysis groups.

We also performed the Kaplan–Meier survival curves for both groups. As already mentioned, in the dialysis group (*n* = 132), there were reported 58 deceased patients (43.94%), and in the non-dialysis group (*n* = 362), 152 deceased patients (41.99%). The median survival value in the dialysis group was 21 days (95% CI of 19 to 42 days), and 26 days (95% CI of 21 to 29 days) in the non-dialysis group (Figure 4).

The curves of survival for the two groups were not significantly different—Chi-square = 0.002356, df = 1, *p* = 0.9613 (Log-rank test). In addition, we obtained an HR of 0.9613 (95% CI of 0.7339 to 1.3426), which represents a measure of how rapidly the event of interest occurs (death). This HR was not significantly different from the value one (corresponding to equal hazards), since the confidence interval included the value one. Therefore, the hazards were equal in both groups.

Furthermore, when assessing the survival proportion in various moments for both groups, it was shown that on day 1, in the dialysis group the survival proportion was 0.985 (98.5%), and 0.015 (1.5%) of patients died. At the same moment, in the non-dialysis group, the survival proportion was 0.997 (99.7%), and 0.003 (0.3%) of patients died (Table S1). As already mentioned, these values showed no significant statistical differences.

## 4. Discussion

Since the outbreak of the COVID-19 pandemic, many studies were conducted in order to determine the profile of COVID-19 patients and the factors that could have an adverse outcome on the evolution of this disease that killed an impressive number of patients all over the world, probably due to the systemic effect of SARS-CoV-2 infection. As already mentioned, it is acknowledged that this systemic effect is a consequence of the modality that this virus has interacting with the host cell: the coronavirus spike binds to angiotensin-converting enzyme 2 (the membrane-bound form) and TMPRSS2 that are found in the lungs, but also in the heart, vessels, kidneys, small intestine epithelium, liver, testicles, ovaries, etc., representing an explanation of COVID-19 multi-system impact [22,23,24].

In 2020, Henry et al.’s systemic review was the first meta-analysis in the literature data that highlighted the possible biomarkers linked to a severe evolution of COVID-19 [25]:

1. Hematologic changes—leukocytosis and neutrophilia, lymphopenia, thrombocytopenia, decreased eosinophil count, and anemia;
2. Biochemical changes—hypoalbuminemia, increased alanine, and aspartate transaminases, total bilirubin, nitrogenous waste products, LDH, creatinine kinase, creatinine kinase-MB, troponin, and myoglobin;
3. Coagulation changes—increased quantitative D-dimer, and prothrombin time;
4. Inflammatory syndrome—increased, CRP, ESR, ferritin, IL-6, IL-8, IL-10, and procalcitonin.

Our results showed the influence of hemoglobin level, LDH activity, and serum creatinine concentration on the risk of mortality in renal impaired patients diagnosed with COVID-19 (the risk was 1.260 times higher in cases of increased hemoglobin levels, 1.165 times higher for elevated serum creatinine concentrations, and 1.003 times higher for increased LDH activity), but failed to notice a link between the chance of death and the rest of the assessed biomarker levels—serum urea (*p* = 0.898), glycemia (*p* = 0.509), glycosylated hemoglobin (*p* = 0.207), IL-6 (*p* = 0.902), CRP (*p* = 0.194), quantitative D-dimer (*p* = 514), procalcitonin, (*p* = 0.891), ferritin (*p* = 0.446), ESR (*p* = 0.173), and fibrinogen. In accordance with our findings, Morell-Garcia et al.’s study found that CKD male patients diagnosed with COVID-19, older than 65 years old, with increased LDH activity upon admission (in addition to hypoalbuminemia), can present a higher risk for a severe course of the disease [26]. Although an important amount of data indicates a strong association between COVID-19 evolution and CRP, D-dimer, ESR, procalcitonin, ferritin concentrations, etc., it should be taken into account that these findings are related to COVID-19 patients in general, regardless of the disease severity. Danwang et al.’s systemic review, based on 31 studies and 16 meta-analyses, concluded that increased levels of aspartate transaminase, serum creatinine, creatinine kinase, and LDH were noticed in severe cases of COVID-19, and elevated LDH activity, and total bilirubin concentration in deceased patients [27]. In 2020, Yan et al. emphasized that augmented levels of LDH per se may be associated with adverse outcomes [28]. It appears that LDH is an important biomarker, especially for severe cases, being in accordance with our results, as our study included moderate and severe COVID-19 patients. These findings regarding the correlation of LDH increased activity to the risk of mortality can be explained by LDH involvement in aerobic glycolysis, playing the role of catalyzer for the transformation of pyruvate to reversible lactate; lactate that is increased in septic conditions or severe pulmonary impairment as in COVID-19—the tissue’s inability to extract oxygen, and consequently lactic acid synthesis is increased, concomitant with LDH activity [29]. Izcovich et al.’s systemic review identified the following potential risk factors for the severity and mortality in COVID-19 with high and moderate certainty of evidence: age, CKD, diabetes, obesity, neoplasia, creatinine level, LDH activity, procalcitonin concentration, D-dimer level, CRP concentration, etc.; related to IL-6 and ESR levels, there was no strong evidence of certainty (similar to our results) [30]. In contrast, in our study, there was no association between diabetes or obesity and the risk of mortality. A possible explanation is that in our study, diabetes appeared to be controlled in both groups (glycosylated hemoglobin mean value of 6.77 ± 1.68% in the non-dialysis group, and of 6.63 ± 1.79% in the dialysis group, respectively). Related to obesity, this information was based exclusively on the diagnosis existent in the discharge medical report, without knowing the body mass index value, and consequently the classes of obesity.

Another interesting finding was that the risk of death was noticed in cases of increased hemoglobin levels (Exp(b) = 1.260), a result in accordance with literature data, which showed the association between higher hemoglobin concentration and the risk of adverse outcomes in CKD patients [31]. Furthermore, some studies indicated that increased concentration of Hb, hematocrit, and red blood cells can be associated with a risk of thrombosis [32]. In addition, it is acknowledged that COVID-19 patients present an increased risk of thrombosis [33]; therefore, patients with moderate to severe forms of COVID-19 and elevated Hb levels, as in our study, are associated with an increased risk of mortality, due to the exponential associated thrombotic risk.

As already mentioned, a relative instantaneous risk of mortality 1.165 times higher was noted in patients with increased serum creatinine concentration. According to different data, even a small increase in serum creatinine level can represent an independent risk factor for CKD progression and mortality [34], and an elevation of 25–49% could be associated with a 3 to 5-fold increase in death risk [35]. Furthermore, Cheng et al.’s study concluded that increased creatinine levels at admission were associated with an in-hospital high risk of mortality [36]. These results suggest the possibility that even a slight increase in creatinine concentration from patients’ usual baseline may represent a predictive factor for the probability of death. Dialysis patients have constant elevated values of serum creatinine. In this kind of patient, serum creatinine values only emphasize the stage of end-stage renal disease, and for our study, could represent the cause of mortality differences between the study groups (43.9% dialysis group vs. 42% non-dialysis group).

A cytokine storm, defined as an overactivation of the immune system inducing systemic inflammation, has been noted in COVID-19 patients, and associated with an increased risk of morbidity and mortality. The onset of this highly impressive immune response is probably caused by an inadequate immune response to the coronavirus [37,38,39]. Furthermore, it is considered that in the early phases of the disease, the innate immune system could even be incriminated in virus replication [37]. According to the literature data, the cytokine storm can be noticed after 7–10 days from the onset of COVID-19 [40]. Similarly, we identified that LOS ≤ 10 days could represent a threshold value—with a sensitivity of 53.81 (95% CI of 46.8 to 60.7) and a specificity of 85.56 (95% CI of 80.9 to 89.4)—which can be associated with an increased risk of mortality. In addition, we noticed that a higher number of the deceased patients presented with LOS ≤ 10 and a grade of pulmonary impairment >25% (45.71% vs. 36.67%), and also the requirement of invasive mechanical ventilation (41.43% vs. 34.29%). These findings related to the length of hospital stay cannot be considered a protective condition per se, as other factors are involved in the progression of the disease: therapy, complications related to the therapy (41.43% required invasive mechanical ventilation), comorbidities, etc., but our results are in accordance with the literature data that highlighted the importance of cytokine storm involvement in the progression of COVID-19 [14].

Most of the existent data related to SARS-CoV-2 infection and renal impairment compare the incidence and outcome between CKD, AKI, kidney-transplanted patients, and those without CKD, and only a few studies are focused only on the survival rate of CKD vs. hemodialysis patients diagnosed with COVID-19. Gasparini et al.’s study reported a similar mortality rate in COVID-19 patients with CKD, acute kidney injury, and end-stage chronic disease [41]. When we performed Kaplan–Meyer analysis, we noticed also a comparable survival proportion in both of the study groups (non-dialysis and dialysis). The same results were noticed in Ozturk et al.’s study that reported a similar mortality rate between CKD stages 3–5 and hemodialysis patients [42]. In contrast, Yang et al.’s study observed a higher rate of in-hospital mortality among the chronic dialysis patients compared to CKD patients in pre-dialysis stages. In addition, the authors reported that age over 65 years represented another risk factor linked to a more severe evolution of COVID-19, both for maintenance dialysis and CKD pre-dialysis patients, but failed to observe the influence of male gender in the dialysis group (only in patients with CKD without dialysis) [43]. According to our results, age and also gender were accepted by our logistic regression models related to the probability of death, in both groups.

An important aspect that should be highlighted is that even if the risk of mortality was higher in our dialysis group vs. non-dialysis patients (Exp(b) = 1.839), the survival proportions were similar in both groups (Chi-square = 0.002356, df = 1, *p* = 0.9613 (Logrank test)).

In contrast with the literature data indicating that obesity and diabetes have an adverse outcome on the evolution of COVID-19 [44,45,46,47], we could not find a clear association between these pathological conditions and the risk of mortality, probably because, as already explained, most of our patients presented with controlled diabetes (in both groups, the glycosylated hemoglobin concentration was around 6%), and we had no data related to the classes of obesity. Over the years, different studies have shown the influence of anemia on glycosylated hemoglobin levels, but recently, Katwal et al. noticed that only moderate to severe anemia could lead to an increase in glycosylated hemoglobin concentration, and not mild anemia [48]. Therefore, considering these data, including the mean values of glycosylated hemoglobin and hemoglobin in both our groups, it was highlighted that our patients presented with controlled diabetes. Furthermore, dialysis patients presented with a significantly decreased Hb concentration compared to the non-dialysis group, which should have led to an increase in glycosylated hemoglobin, and as our results showed, even in this case the mean values ranged in the level interval specific for controlled diabetes in CKD patients.

Nevertheless, based on all these findings and implications of this disease on health impairment, it should be highlighted that COVID-19 patients, especially those associated with chronic renal impairment, may present, even after discharge, with persisting multisystem anomalies, requiring further investigations (lab analysis, imagistic tests, etc.) with a direct socio-economic impact on the healthcare system [49].

## 5. Limitations

Our study had some limitations, as the diagnosis of CKD was exclusively based on the diagnosis existing in the patients’ discharge medical records, and not classified according to the estimated glomerular filtration rate. Therefore, we could not make a difference between the CKD stages. Similarly, for obesity, the diagnosis was based on the discharge medical records, and not on the level of body mass index that was not available in the assessed medical records. Even if we included patients with kidney transplantation and peritoneal dialysis, the number was too small in order to be able to perform a valid statistical analysis, and therefore, these patients were assimilated into the non-dialysis group or dialysis group, respectively. Similarly, the number of vaccinated patients was insufficient in order to perform further relevant statistical analysis. In addition, even if all patients received our standard national protocol regimen for infection with SARS-CoV-2 as stipulated by the Romanian Health Ministry, we could not indicate the customized therapy for each patient, as this information was not available in the informatic database of the hospital. Regarding the serum creatinine concentration, whether it represented a pre- or post-dialysis value, this information was not available for all patients in our informatic database system, so we could not make an assessment.

## 6. Conclusions

The COVID-19 pandemic has changed our perspective of life and made us more attentive regarding the importance of having an adequate health care system, and of better control and management of various preexisting prophylaxis conditions. As the literature data show, chronic kidney disease patients (on maintenance dialysis or not), especially the elderly, are more susceptible to being associated with severe forms of COVID-19, presenting a significant rate of mortality, as well. Several explanations are reported, including the correlation with a more dramatic inadequate innate immune response in this population group, highlighted by the presence of several increased biomarkers, such as increased LDH activity, procalcitonin level, ESR concentration, CRP level, hypoalbuminemia, etc. Our study concluded that high levels of hemoglobin, serum creatinine, and LDH at admission could be linked to the probability of death. In addition, age, LOS less than 10 days, and a pulmonary impairment higher than 25% are responsible for an adverse outcome in non-dialysis and dialysis patients diagnosed with COVID-19. No association between diabetes or obesity and mortality was noticed in our patients. Additionally, the proportion of survival was similar for non-dialysis and dialysis patients. The available information regarding chronic renal impaired patients with SARS-CoV-2 infection seems to be ununified, and probably larger trials are required to be performed in order to validate these results and to fill in the missing data.

## Figures and Tables

**Table 1:** Assessed parameters at the time of admission (performed method and laboratory device).

Parameter	Normal Value Range	Assay	Laboratory Device
Hemoglobin	12.3–17 g/dL	Photometric method, analyzed using HBG-photometric detection, following SLS hemoglobin chamber	SYSMEX XN-2000-HLG-5diff
Serum creatinine	0.7–1.2 mg/dL	Jaffe method (the colorimetric technique)	COBAS 501 (Roche)
Serum urea	17.4–49 mg/dL	Urease method	COBAS 501 (Roche)
Glycemia	80–115 mg/dL	Hexokinase method	COBAS 501 (Roche)
Glycosylated hemoglobin	4.8–5.6%	Turbidimetric method	COBAS 501 (Roche)
Interleukin-6 (IL-6)	<7 pg/mL	Electrochemiluminescence (ECLIA) method	COBAS 601 (Roche)
C-reactive protein (CRP)	≤5 mg/L	Turbidimetric method	COBAS 501 (Roche)
Lactate dehydrogenase (LDH)	135–225 UI/L	Ultraviolet method (with pyruvate)	COBAS 501 (Roche)
Serum albumin	3.4–5.2 g/dL	Colorimetric method	COBAS 501 (Roche)
Serum total proteins	6.4–8.3 g/dL	Colorimetric method	COBAS 501 (Roche)
Quantitative D-dimer	0–0.5 μg/mL	Immunoturbidimetric method	STA_R_ Max 2—STAGO Top Diagnostics
Procalcitonin	≤0.05 ng/mL	Electrochemiluminescence (ECLIA) method	COBAS 601 (Roche)
Ferritin	30–400 ng/mL	Electrochemiluminescence (ECLIA) method	COBAS 601 (Roche)
Erythrocyte sedimentation rate (ESR)	2–20 mm/h	Capillary microphotometry (automatic method)	ALIFAX
Fibrinogen	200–400 mg/dL	Mechanical method to determine fibrinogen concentration (measurement of the conversion of fibrinogen to fibrin, in the presence of excess thrombin)	STA_R_ Max 2—STAGO Top Diagnostics

**Table 2:** Patients’ characteristics in both groups.

Data		Non-Dialysis Group (*n* = 362)	Dialysis Group (*n* = 132)	*p* Value
Age (years; mean ± SD values)		72.56 ± 13.10	64.89 ± 12.07	<0.001
Gender	male	213 (58.8%)	81 (61.4%)	0.613
	female	149 (41.2%)	51 (38.6%)	
Length of hospital stay (days; mean ± SD values)		15.24 ± 9.70	15.82 ± 9.78	0.557
Discharge status	Discharge	183 (50.6%)	57 (43.2%)	0.207
	Deceased	152 (42%)	58 (43.9%)	
	Transferred to another hospital	11 (3%)	8 (6.1%)	
	Discharge by request	16 (4.4%)	9 (6.8%)	
Patient’s environment	Urban	280 (77.3%)	100 (75.8%)	0.710
	Rural	82 (22.7%)	32 (24.2%)	
Medical departments (patients’ admission)	Nephrology	155 (42.8%)	123 (93.2%)	<0.001
	Urology	34 (9.4%)	3 (2.3%)	0.0183
	Cardiology	83 (22.9%)	2 (1.5%)	<0.001
	Internal medicine	31 (8.6%)	0 (0%)	<0.001
	Vascular surgery	8 (2.2%)	0 (0%)	0.086
	Plastic surgery	10 (2.8%)	0 (0%)	0.052
	Gastroenterology	23 (6.4%)	1 (0.8%)	0.01
	Orthopedy	5 (1.4%)	0 (0%)	0.172
	General surgery	11 (3%)	3 (2.3%)	0.677
	Thoracic surgery	1 (0.3%)	0 (0%)	0.529
	Gynecology	1 (0.3%)	0 (0%)	0.529
Diabetes mellitus		178 (49.2%)	52 (39.4%)	0.054
Obesity		141 (39%)	49 (37.1%)	0.712
Grade of pulmonary impairment	without	27 (7.5%)	14 (10.6%)	0.270
	≤25%	122 (33.7%)	44 (33.3%)	0.933
	26–50%	85 (23.5%)	26 (19.7%)	0.371
	51–75%	74 (20.4%)	26 (19.7%)	0.864
	76–100%	54 (14.9%)	22 (16.7%)	0.624
Oxygen therapy	High flow oxygen therapy (AIRVO)	7 (1.9%)	1 (0.8%)	0.359
	Invasive mechanical ventilation	143 (39.5%)	53 (40.2%)	0.896
	Non-invasive mechanical ventilation	52 (14.4%)	29 (22%)	0.043
Admission hemoglobin (g/dL; mean ± SD values)		11.64 ± 2.64	9.91 ± 1.96	<0.001
Admission serum creatinine (mg/dL; mean ± SD values)		3.64 ± 3.56	8.23 ± 3.23	<0.001
Admission serum urea (mg/dL; mean ± SD values)		141.49 ± 89.93	155.82 ± 81.89	0.109
Admission glycemia (mg/dL; mean ± SD values)		157.12 ± 88.49	145.31 ± 88.37	0.190
Admission glycosylated hemoglobin (%; mean ± SD values)		6.77 ± 1.68	6.63 ± 1.79	0.552
Admission IL-6 (pg/mL; mean ± SD values)		216 ± 661.08	231.99 ± 472.38	0.001
Admission CRP (mg/L; mean ± SD values)		112.21 ± 98.13	123.91 ± 105.23	0.254
Admission LDH (UI/L; mean ± SD values)		428.50 ± 258.58	422.89 ± 276.29	0.839
Admission serum albumin (g/dL; mean ± SD values)		3.36 ± 0.59	3.51 ± 0.53	0.016
Admission serum total proteins (g/dL; mean ± SD values)		6.49 ± 0.86	6.62 ± 0.79	0.168
Admission quantitative D-dimer (μg/mL; mean ± SD values)		2.71 ± 2.69	2.93 ± 2.77	0.117
Admission procalcitonin (ng/mL; mean ± SD values)		3.29 ± 12.85	7.32 ± 19.52	0.002
Admission ferritin (ng/mL; mean ± SD values)		1352.26 ± 1499.85	2213.20 ± 2327.83	<0.001
Admission ESR (mm/h; mean ± SD values)		66.74 ± 31.22	72.31 ± 29.30	0.083
Admission fibrinogen (mg/dL; mean ± SD values)		602.68 ± 184.38	585 ± 182.10	0.349

Notes: All the results are conferred as numbers and percentages, or as mean ± SD. CRP—C-reactive protein; ESR—erythrocyte sedimentation rate; IL-6—interleukin-6; LDH—lactate dehydrogenase; SD—standard deviation.

**Table 3:** The relative instantaneous risks of mortality in the dialysis group.

Variables	b	S.E.	Wald	df	*p*	Exp(b)	95% CI of Exp(b)
Age (years)	0.049	0.010	25.365	1	<0.001	1.051	1.031 to 1.071
LOS (≤10 days)	2.027	0.256	62.566	1	<0.001	7.590	4.593 to 12.541
Gender (male)	0.496	0.234	4.506	1	0.034	1.642	1.039 to 2.595
Grade of pulmonary impairment (>25%)	2.038	0.250	66.607	1	<0.001	7.675	4.704 to 12.520
Dialysis group	0.609	0.269	5.131	1	0.023	1.839	1.086 to 3.116

Notes: The table lists the variables included in the model, their regression coefficient b with standard error (SE), Wald statistic (b/SE)2 and associated *p*-value, Exp(b) and the 95% confidence interval for (Exp(b)).

**Table 4:** The relative instantaneous risks of mortality in the non-dialysis group.

Variables	b	S.E.	Wald	df	*p*	Exp(b)	95% CI of Exp(b)
Age (years)	0.049	0.010	25.365	1	<0.001	1.051	1.031 to 1.071
LOS (≤10 days)	2.027	0.256	62.566	1	<0.001	7.590	4.593 to 12.541
Gender (male)	0.496	0.234	4.506	1	0.034	1.642	1.039 to 2.595
Grade of pulmonary impairment (>25%)	2.038	0.250	66.607	1	<0.001	7.675	4.704 to 12.520
Non-dialysis group	−0.609	0.269	5.131	1	0.023	0.544	0.321 to 0.921

Notes: The table lists the variables included in the model, their regression coefficient b with standard error (SE), Wald statistic (b/SE)2 and associated *p*-value, Exp(b) and the 95% confidence interval for (Exp(b)).

**Table 5:** The risk of mortality in a dialysis male patient.

Age (Years)	Logit (*p*)	*p*
30	0.435	0.6071
35	0.680	0.6637
40	0.925	0.7161
45	1.170	0.7631
50	1.415	0.8046
55	1.660	0.8402
60	1.905	0.8705
65	2.150	0.8957
70	2.395	0.9164
75	2.640	0.9334
80	2.885	0.9471

Notes: *p*—the probability of death according to age for a dialysis male patient presenting LOS ≤ 10 days, and a grade of pulmonary impairment >25%.

**Table 6:** The risk of mortality in a non-dialysis male patient.

Age (Years)	Logit (*p*)	*p*
30	−0.159	0.4603
35	0.088	0.5220
40	0.336	0.5831
45	0.583	0.6418
50	0.831	0.6965
55	1.078	0.7461
60	1.325	0.7901
65	1.573	0.8282
70	1.820	0.8606
75	2.068	0.8877
80	2.315	0.9101

Notes: *p*—the probability of death according to age for a non-dialysis male patient presenting LOS ≤10 days, and a grade of pulmonary impairment >25%.

**Table 7:** The relative instantaneous risks of mortality.

Variables	b	S.E.	Wald	df	*p*	Exp(b)	95% CI of Exp(b)
Age (years)	0.047	0.015	10.367	1	0.001	1.048	1.019 to 1.079
LOS (≤10 days)	2.365	0.392	36.387	1	<0.001	10.643	4.936 to 22.950
Hb	0.231	0.084	7.459	1	0.006	1.260	1.067 to 1.486
Serum creatinine	0.153	0.054	8.041	1	0.005	1.165	1.048 to 1.295
LDH	0.003	0.001	10.829	1	0.001	1.003	1.001 to 1.004

Notes: The table lists the variables included in the model, their regression coefficient b with standard error (SE), Wald statistic (b/SE)2 and associated *p*-value, Exp(b) and the 95% confidence interval for (Exp(b)). The listed biomarkers were assessed at the admission. Hb—hemoglobin; LDH—lactate dehydrogenase.

## Data Availability

Data can be found in the electronic database of “St. John” Emergency Clinical Hospital, Bucharest, Romania, which cannot be publicly disclosed, being subjected to GDPR data protection policy and fines.

## Supplementary Materials

The following are available online at https://www.mdpi.com/article/10.3390/jpm12060966/s1, Table S1: The survival proportion in both groups.
