# Supplementary material for: Epidemiological Characteristics and Mortality Risk Factors Comparison in Dialysis and Non-Dialysis CKD Patients with COVID-19—A Single Center Experience
Source: J Pers Med. 2022 Jun 13;12(6):966. doi: 10.3390/jpm12060966 (PMC9224649; doi:10.3390/jpm12060966)
Supplement: Supplementary file 1 [file jpm-12-00966-s001.zip › jpm-1750956-supplementary.pdf]

Supplementary Table S1. The survival proportion in both groups.

Day	Dialysis group		Non-dialysis group		Overall	
	Survival proportion	Standard error	Survival proportion	Standard error	Survival proportion	Standard error
1	0.985	0.0106	0.997	0.00276	0.994	0.00350
2	0.939	0.0208	0.972	0.00864	0.964	0.00844
3	0.932	0.0219	0.922	0.0141	0.925	0.0119
4	0.894	0.0269	0.889	0.0166	0.890	0.0141
5	0.886	0.0277	0.872	0.0176	0.876	0.0149
6	0.855	0.0309	0.852	0.0187	0.853	0.0160
7	0.823	0.0335	0.819	0.0204	0.820	0.0174
8	0.783	0.0363	0.807	0.0209	0.801	0.0181
9	0.775	0.0368	0.787	0.0217	0.784	0.0187
10	0.759	0.0378	0.769	0.0224	0.766	0.0193
15	0.685	0.0423	0.682	0.0256	0.683	0.0219
20	0.538	0.0516	0.570	0.0308	0.561	0.0265
25	0.450	0.0566	0.511	0.0348	0.492	0.0299
30	0.450	0.0566	0.362	0.0456	0.387	0.0371
45	0.281	0.102	0.259	0.0570	0.264	0.0503
